# Detection of genome-wide structural variations in the Shanghai Holstein cattle population using next-generation sequencing

**DOI:** 10.5713/ajas.18.0204

**Published:** 2018-07-26

**Authors:** Dengying Liu, Zhenliang Chen, Zhe Zhang, Hao Sun, Peipei Ma, Kai Zhu, Guanglei Liu, Qishan Wang, Yuchun Pan

**Affiliations:** 1 Department of Animal Science, School of Agriculture and Biology, Shanghai Jiao Tong University, Shanghai 200240, China; 2 Shanghai Key Laboratory of Veterinary Biotechnology, Shanghai 200240, China; 3 Shanghai Dairy Cattle Breeding Centre Co., Ltd, Shanghai 201901, China

**Keywords:** Genotyping by Genome Reducing and Sequencing (GGRS), Mastitis, Reproduction, Shanghai Holstein, Structural Variation

## Abstract

**Objective:**

The Shanghai Holstein cattle breed is susceptible to severe mastitis and other diseases due to the hot weather and long-term humidity in Shanghai, which is the main distribution centre for providing Holstein semen to various farms throughout China. Our objective was to determine the genetic mechanisms influencing economically important traits, especially diseases that have huge impact on the yield and quality of milk as well as reproduction.

**Methods:**

In our study, we detected the structural variations of 1,092 Shanghai Holstein cows by using next-generation sequencing. We used the DELLY software to identify deletions and insertions, cn.MOPS to identify copy-number variants (CNVs). Furthermore, we annotated these structural variations using different bioinformatics tools, such as gene ontology, cattle quantitative trait locus (QTL) database and ingenuity pathway analysis (IPA).

**Results:**

The average number of high-quality reads was 3,046,279. After filtering, a total of 16,831 deletions, 12,735 insertions and 490 CNVs were identified. The annotation results showed that these mapped genes were significantly enriched for specific biological functions, such as disease and reproduction. In addition, the enrichment results based on the cattle QTL database showed that the number of variants related to milk and reproduction was higher than the number of variants related to other traits. IPA core analysis found that the structural variations were related to reproduction, lipid metabolism, and inflammation. According to the functional analysis, structural variations were important factors affecting the variation of different traits in Shanghai Holstein cattle. Our results provide meaningful information about structural variations, which may be useful in future assessments of the associations between variations and important phenotypes in Shanghai Holstein cattle.

**Conclusion:**

Structural variations identified in this study were extremely different from those of previous studies. Many structural variations were found to be associated with mastitis and reproductive system diseases; these results are in accordance with the characteristics of the environment that Shanghai Holstein cattle experience.

## INTRODUCTION

Holstein cows are famous for having the highest milk yield in the world; therefore, much attention has shifted to the breeding of Holstein cattle for many years [1]. Chinese Holstein is the main dairy cattle breed in China, and the semen of their bulls are used in approximately 20% of the whole country. Therefore, it is important to conduct studies to improve the breeding value of bulls in Shanghai to further enhance the performance of Holstein cows in China.

The Holstein breed is categorized as a heat-sensitive cattle breed that originated in Europe. High environmental temperatures have a negative influence on the performance of Chinese Holstein cattle, especially in Shanghai [2,3]. The living environment of Shanghai Holstein cattle is characterized by a very hot temperatures especially in the summer and long-term humidity, which causes high susceptibility to diseases such as mastitis resulting in a decrease in the yield and quality of milk as well as huge influence on reproduction thereby causing abortions and stillbirths [3–6].

Despite this poor natural habitat, it has been long observed and established that Shanghai Holstein cattle are less susceptible to mastitis. Livestock breeds of Shanghai origin are well adapted to tropical environments because of their ability to thrive under extreme nutritional stress, resistance to diseases, heat tolerance potential and sturdiness [5,7,8]. Therefore, there has been high interest in investigating the factors that might affect milk quality as well as cause disease at the molecular level. Previous reports found that structural variations influenced the performance of Holstein cattle directly or indirectly.

Genetic variation ranges from the single base pair (bp) to several megabases (Mb) compared to a reference genome [9]. As a type of genetic variation, structural variation, including gains and losses of DNA segments and balanced rearrangements, often refers to large-scale structural differences in the genomic DNA that is inherited and polymorphic in a species. Structural variation was originally defined as insertions, deletions and large-scale copy-number variants (CNVs) with a size greater than 1 kb [10–13]. A significant amount of research has redefined the spectrum of structural variation as variants with a length >50 bp [14–16]. Many studies indicated that structural variations have been associated with a variety of diseases in humans and other species, particularly cancer, autism, schizophrenia, and neurodevelopmental disorders, by altering gene dosage and/or disrupting genes in the form of deletions or duplications [9,17–22]. In addition, structural variations cause increases in the cost of livestock by affecting economic traits, e.g., deletions in MER1 repeat containing imprinted transcript 1 resulted in abortions and stillbirths [23]. The impact of structural variation on traits is significantly greater than single nucleotide polymorphisms (SNPs), and the percentages of their contribution to complex phenotypes are 83.6% and 17.7%, respectively [14]. CNVs, as one of the main types of genomic structural variation, can be considered to be promising causal markers for some traits [19]. However, the genome-wide structural variation of Shanghai Holstein cattle has rarely been reported.

Therefore, the objective of this study was to identify the structural variants in the Shanghai Holstein population and determine the genetic mechanism of the influence of structural variation on the performance of Shanghai Holstein cattle by annotating the genome for the distribution, density and physical location of the main structural variations on chromosomes.

## MATERIALS AND METHODS

### Ethics statements

All experimental procedures were approved by the Institutional Animal Care and Use Committee of Shanghai Jiao Tong University, and all methods involving cattle were in accordance with the agreement of Institutional Animal Care and Use Committee of Shanghai Jiao Tong University (contract no. 2011-0033).

### Animals

The Holstein population in this study comprised 1,092 cows, the daughters of 17 sires and each family with an average of 64. These cows where born between 2001 and 2012. All the cows where from 24 dairy cattle farms in the Shanghai Bright Dairy and Food Co., Ltd where have been carried out as part of the Dairy Herd Improvement System.

### DNA and sequencing data collection

The DNA samples were genotyped according to the genotyping by genome reducing and sequencing (GGRS) protocol [24]. Briefly, high molecular weight genomic DNA was extracted from blood samples using the Multisource Genomic DNA Extraction Kit (Axygen Biotechnology Co., Ltd, Hangzhou, China) and then digested with *AvaII*, followed by ligation with a unique adapter-barcode sequence for *AvaII* (5′ACACTC TTTCCCTACACGACGCTCTTCCGATCTXXXXX3′ and 5′GWCYYYYYAGATCGGAAGAGCGGTTCAGCAGGAA TGCCGAG3′, where XXXXX and YYYYY denote the barcode and the reverse barcode complementary sequences, respectively). Next, 84 or 108 samples were pooled and enriched by polymerase chain reaction amplification (Primer1.1, 5′AATG ATACGGCGACCACCGAGATCTACACTCTTTCCCTAC ACGACGCTCTTCCGATCT; Primer2.1, 5′CAAGCAGAAGA CGGCATACGAGATCGGTCTCGGCATTCCTGCTGAAC CGCTCTTCCGATCT). The quality of the sequencing libraries was evaluated by an Agilent 2100 Bioanalyzer (GENEWIZ, Suzhou, China). Lastly, sequencing libraries (fragments ranging from 300 to 400 bp, including adapter-barcode sequences) were sequenced on an Illumina HiSeq2000 (Illumina, Inc., San Diego, CA, USA) instrument to obtain paired-end (2×150 bp) reads. After sequencing, the sequence reads were filtered for quality using the NGSQC Toolkit v2.3 with the parameter settings referred to in Chen et al [24] and then aligned to the UMD3.1 reference sequence using the Burrows-Wheeler Aligner (BWA ver 0.7.5), with the default settings and the steps outlined in the GGRS approach [24]. In addition, we excluded reads that could not be mapped or were mapped to the Mitochondrial and Y chromosomes because those reads had very high rates of discordance between sires and progenies [7,25,26].

### Structural variation measurements and filtration

In our research, our objective was to identify the variations in DNA sequences of cattle from southern China, especially deletions, insertions and CNVs. Deletions and insertions were called using paired-end mapping, and CNVs were called using single-end mapping. The most important difference between these mapping strategies is that with high-quality mapping of reads in regions with repeat content, single-end reads are unfortunately unsuitable to accurately predict transcription in repeat-containing regions. Deletions, insertions and CNVs were analysed in this study. DELLY (version 0.5.9) used to call deletions and insertions with paired-end mapping data and the default settings of the analysis parameters is based on multiple samples and uses discordant reads to find candidate SVs and then verifies the exact breakpoints by split-read alignments [27]. After deletions or insertions were detected, we chose deletions or insertions with more than three paired-end reads, a length between 300 bp and 1,000 bp, and a mapping quality equal to or greater than 30 [28]. We used the cn.MOPS algorithm to detect CNVs, which are defined as unbalanced structural variants that change the number of base pairs in the genome. cn.MOPS is proposed to increase statistical power and decrease computational burden based on a multiple samples approach. cn.MOPS proposed a data processing pipeline using a mixture of Poisson models to reduce the false discovery rate (FDR) in CNV detection. In addition, we treated structural variations as a CNV when changes with a length of more than 1 kb were identified in at least one sample and were not in an amphiploid form [29].

### Structure and function annotation

We annotated the structural variations using different bioinformatics tools, including the database for annotation, visualization and integrated discovery (DAVID), ingenuity pathway analysis (IPA), and the cattle quantitative trait locus (QTL) database (cattle QTLdb). First, we expected to determine the location or distance distribution and density of variants across the chromosomes by downloading the Ensembl bovine gene annotation set (Ensembl release 78) from the Ensembl website (ftp://ftp.ensembl.org/pub/) and then use Perl or R tools to complete the matches. For our analyses, we selected the gene IDs of Ensembl that contained at least one variant. Then, at the level of function, we continued to refine our annotations using DAVID v6.8 (https://david.ncifcrf.gov/summary.jsp), which consists of an integrated biological knowledgebase and analytic tools aimed at systematically extracting biological meaning from large gene/protein lists [30]. The list of genes containing variants was subjected to pathway analysis using DAVID. The Kyoto encyclopedia of genes and genomes (KEGG) pathway and gene ontology (GO) analyses were performed on 3,164 genes whose sequences overlapped with deletions, 2,537 genes whose sequences overlapped with insertions and 207 genes that overlapped with CNVs. The enriched GO terms and KEGG pathways with p values <0.05 after correction for multiple testing were considered to be statistically significant. The IPA is a web-based functional analysis tool for comprehensive omics data. At present, the IPA software has enabled great progress in systematic bioinformatics analyses, allowing us to better interpret gene expression profiles [31]. We uploaded the gene list that overlapped with our results to Qiagen’s IPA system for core analysis; the numbers of genes with deletions, insertions and CNVs were 3,178, 2,542, and 192, respectively. In our study, IPA analysis was performed to identify canonical pathways, disease and functions, and gene networks there were most significant to our outcomes and to categorize differentially expressed gene into specific diseases and functions, such as mastitis and reproductive disorders in Holstein cattle [32].

Another cost-effective approach to compare, confirm, and locate the most plausible location of genes related to important traits is to align our results with the QTLdb of UMD_3.1, which contains 95,332 QTLs/associations (http://www.animalgenome.org/cgi-bin/QTLdb/BT/index, updated Sept. 2016). We identified all the QTLs that contained or overlapped with the variants. After matching, the number and function of variants was identified, and these variants were used for subsequent analyses.

## RESULTS

### Sequencing data analysis

The 1,092 samples were divided into 12 libraries. The 1st and 2nd libraries are two replicates of the same individuals, so they were combined in the following analyses. A total of 41 million qualified reads were generated by Illumina HiSeq2000. The sequencing quality of libraries with 84 and 108 individuals are listed in Supplementary Figure S1, which shows the sequencing quality of two libraries as an example. We found that the average Phred score for each position was greater than 20 after removing the primer/adapter-contaminated reads. Furthermore, after sequencing, we conducted quality control by using the NGSQC package (NGSQCToolkit_v2.3.3). The average number of qualified reads generated by Illumina HiSeq2000 for the 12 pools was 3,046,279, and the average depth and coverage for SNP calling were 4.0% and 1.8%, respectively. Detailed information is shown in Table 1.

### Characterization of variants

**Figure 1:**
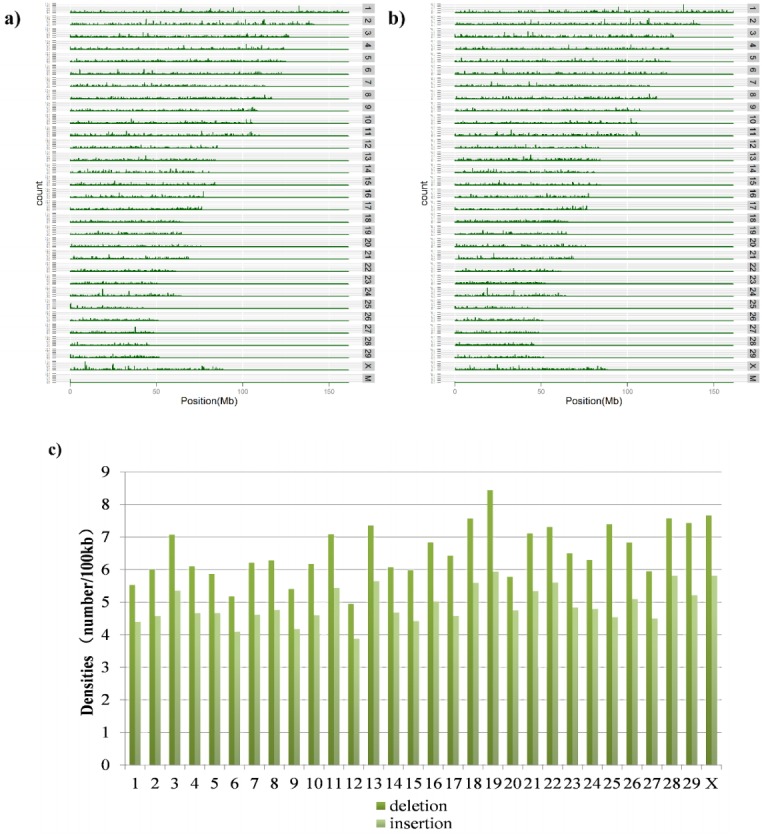
Distribution of the density of deletions and insertions across chromosomes. (a) Shows the location distribution of deletions across the genome; (b) represents the location distribution of insertions across the genome; and (c) demonstrates the distribution of the density of deletions and insertions across chromosomes calculated as the number of structure variations per 100 kb.

A total of 16,831 deletions, 12,735 insertions and 490 CNVs were identified from the sequence data of the Shanghai Holstein population. The distribution of the number and density of deletions and insertions on the 29 autosomes and X chromosome is shown in Figure 1, and the distribution for CNVs is shown in Supplementary Figure S2. In general, the number of deletions detected in each chromosome was much more than the number of insertions and CNVs. The density distributions of deletions and insertions were relatively similar and quite high on chromosomes 18, 19, and 28. Chromosome 19 had the highest density, while chromosome 12 had the lowest density, among all the chromosomes. The average length of the deletions and insertions was approximately 340 bp. In addition, the distribution on each chromosome for different types of structural variations except CNVs is quite uniform. Furthermore, in our study, the average number of CNVs across the 30 chromosomes was 16. The density of CNVs on chromosomes 14 and 28 was the lowest (0.6%), whereas, it was the highest on chromosome 25 (7%). The length of CNVs was between 1 kb and 200 kb; the length of most CNVs (56.12%) was between 1 kb and 10 kb; and the average length of CNVs was 13,725 bp. There were 450 CNV regions (CNVRs). The number and length of CNVRs were also distributed randomly. Chromosome 3 is the longest of all the chromosomes, and it had the fewest CNVRs, while chromosome 25 is the shortest but had the most CNVRs (Supplementary Table S1).

To further explore the distribution of variants in genic regions (intergenic, exonic, intronic, and untranslated regions), we annotated all detected variants using the Ensembl gene set (containing 24,616 genes). The results are shown in Table 2. Within genic regions, there were 5,168 (30.71%), 3,852 (30.25%), and 211 (46.89%) identified deletions, insertions and CNVRs, respectively. In addition, the number of total genes for deletions, insertions and CNVs contained in the Ensembl gene database of bovine are 3,160 (13.65%), 2,532 (11.07%), and 239. It was observed that most CNVRs were overlapping with only a single gene, and the CNV on chr2:90,562,215 – 90,665,303, overlapped with 4 genes. The range of the number of deletions relative to genes distributed on different chromosomes was from 5.85% to 19.44%; and for insertions, the range was from 5.23% to 16.14%. Chromosomes 24, 26, and 28 had the highest number of genes with deletions and insertions.

To assess the function of structural variants accurately, we investigated the distribution of the variants in each type of genomic location, which provided a powerful approach for understanding the potential roles of variants in functional regions. Although the majority of the deletions (11,662, 69.29%) were located in intergenic regions, only approximately 4.09% (689) of them were located in exonic regions. Compared to the distribution of deletions, the distribution of insertions had a similar result (8,882, 69.74% in intergenic regions; 471, 3.70% in exonic regions). In addition, 56.94% (279) of CNVs were located in intergenic regions and 29.18% (143) were in exonic regions, where there are the most variations (Table 3).

### Alignment to the QTL database

**Figure 2:**
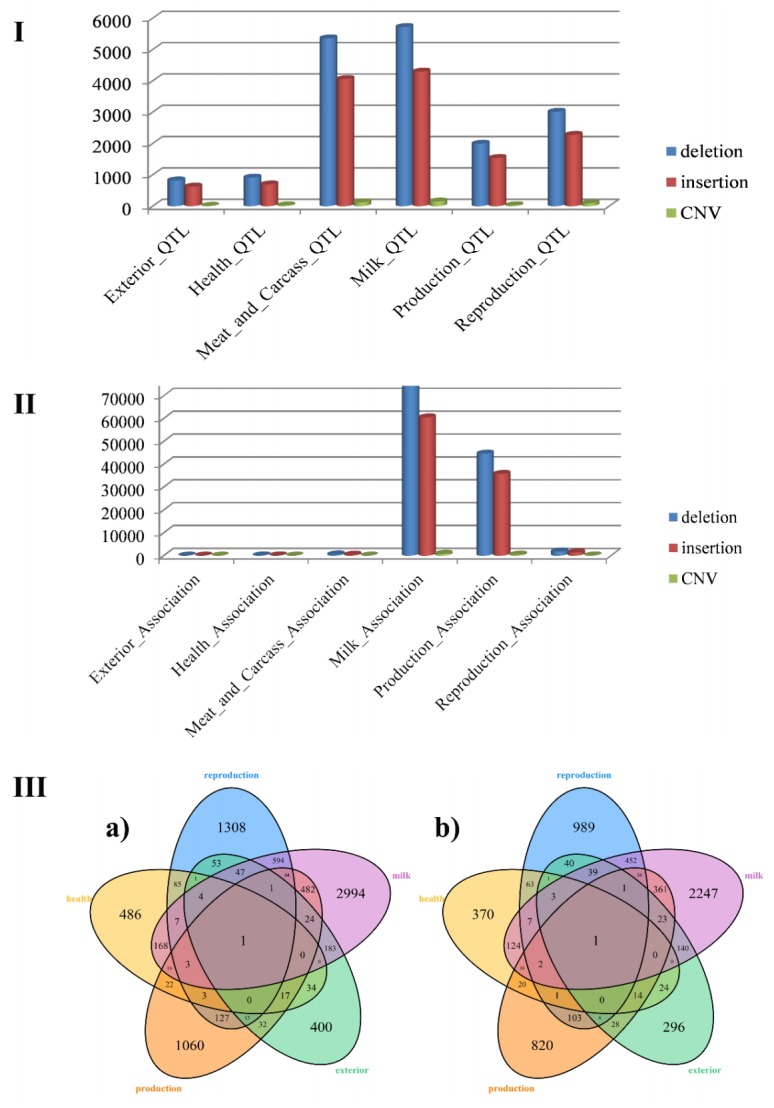
The proportion of distributions of variants overlapping quantitative trait loci (QTLs) and Ensembl genes for six traits and Venn diagrams of deletions and insertions among different traits. I. Shows the distribution of different traits with different structure variations associated with Exterior_QTL, Health_QTL, Meat_and_Carcass_QTL, Milk_QTL, Production_QTL and Reproduction_QTL; II. Shows the distribution of different traits with different structure variations associated with Exterior_Association, Health_Association, Meat_and_Carcass_Association, Milk_Association, Production_Association and Reproduction_Association; III. Shows the deletion (a) and insertion (b) number distribution of different traits.

It is necessary to detect all the variants that are contained in or overlap with QTLs, as QTL detection provides valuable information to describe functionally important variants and to understand genetic mechanisms underlying dairy phenotypes. Hence, the variants directly linked to complex traits could be determined. Six traits (exterior, health, meat and carcass quality, milk, production and reproduction) were analysed in our study. All the variants identified were aligned to QTLs based on the cattle QTL database using location information. The results showed that a structural variant might overlap with several QTLs linked to different traits, and we kept all QTLs in further analyses. There were 140,289 deletions, 11,694 insertions and 1,829 CNVs that overlapped QTLs (Table 4). Overall, we determined that the distributions of all types of variants presented the same pattern, in which the number of variants related to milk was higher than the number of variants related to other traits In contrast, the number of variants related to exterior was the lowest (Figure 2). According to systematic analysis, the proportion of deletions for each trait was 4.63% (exterior), 5.13% (health), 30.05% (meat and carcass quality), 32.08% (milk), 11.19% (production), and 16.92% (reproduction), and the proportion of insertions for each trait was 4.65%, 5.17%, 30.06%, 31.83%, 11.40%, and 16.89%, respectively, and the proportion of CNVs for each trait was 3.79%, 7.16%, 26.53%, 33.68%, 8.21%, and 20.63%, respectively.

Then, we detected the distribution of variants in both QTLdb and Ensembl, and the pattern of distribution is quite similar to that of traits located in only QTLdb. There were 39,546 deletions, 29,881 insertions, and 689 CNVs located in QTLdb. The trait with the highest percentage of variants located in both QTLdb and Ensembl was milk, which was consistent with the results of the variants distributed in only QTLdb. Because the size of a QTL is quite large, it reflects different fragments other than a single site or gene that influences quantitative traits.

To determine how many structural variations are associated with different traits, an R script was used to draw Venn diagrams (Figure 2). It is interesting that 168 deletions, which comprise 34.57% of the total related to health, were also associated with milk, and 124 of 370 insertions related to health were also associated with milk. We also found that approximately 45.47% of the deletions related to production overlapped with QTLs related to milk, and a similar pattern was found for insertions.

### Gene ontology enrichment and pathway analysis

A large fraction of the bovine genes were assigned to categories and pathways in the GO and KEGG databases. Therefore, GO and KEGG databases were used to identify enriched biological functions and to analyse the role that variants play in the regulatory networks in this study. The gene list of deletions, insertions and CNVs were uploaded to DAVID v6.8. There were 525, 369, and 126 genes left to cluster after removing repeat or unknown genes. After filtering for variants with p values <0.05, functional annotations of these deletions and insertions identified 16 and 12 KEGG pathways, and 26 and 31 GO terms, for both deletions and insertions respectively. Because the number of CNVs was small, we selected the top 17 GO terms and KEGG pathways, including 15 GO terms and 2 pathways (Supplementary Table S2). The key features in GO terms and pathways were different for different types of variants. KEGG pathway analysis indicated that the genes with deletions were involved in 12 KEGG pathways, and genes containing insertions were involved in 16 KEGG pathways. We found that they shared 8 KEGG pathways (bta05200: Pathways in cancer; bta04976:Bile secretion; bta04810:Regulation of actin cytoskeleton; bta04611:Platelet activation; bta04510: Focal adhesion; bta04110:Cell cycle; bta03410:Base excision repair; bta02010:ABC transporters), and 19 GO terms, in which 5 GO terms were also shared by CNVs (GO:0097367~ carbohydrate derivative binding; GO:0044422~organelle part; GO: 0043228~non-membrane-bounded organelle; GO:0036094~ small molecule binding; GO:0022402~cell cycle process). Two GO terms were only shared by deletions and CNVs (GO: 0044463~cell projection part and GO:0043234~protein complex).

The most significant pathway of both deletions and insertions was bat05200:Pathways in cancers; it is worth noting that deletions and insertions were very likely associated with disease resistance in the Shanghai Holstein population. In addition, for functional annotations of CNVs, we found that CNVs mainly clustered in bta00240:Pyrimidine metabolism, bta00240:Pyrimidine metabolism and GO:0043234~neuron part.

### IPA analysis

**Figure 3:**
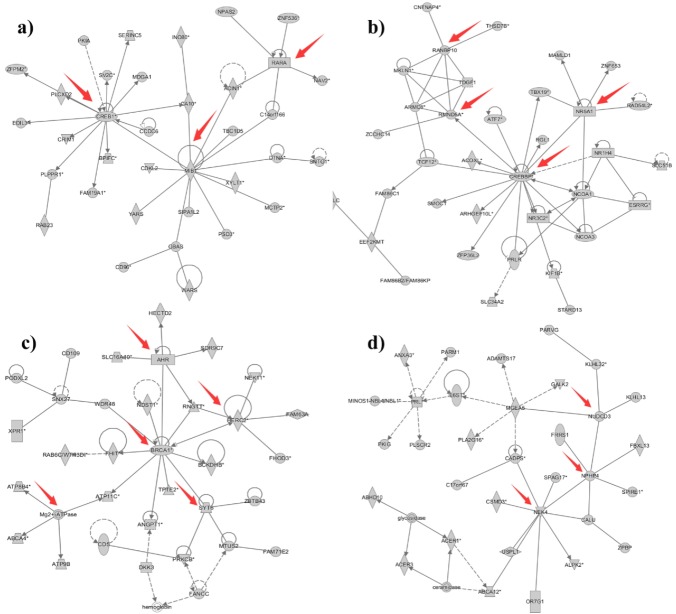
The detailed networks related to different diseases and functions. (a) Shows the network of genes significantly related to embryonic development; (b) shows the network significantly associated with reproductive system development and function; (c) and (d) show the networks significantly related to lipid metabolism. Solid shapes represent the genes in the original module, hollow shapes represent the genes or biomolecules that were added to the network from the ingenuity pathway analysis database to complete the whole network, solid lines show direct interactions between genes, and dashed lines show indirect interactions between genes.

To perform an elementary investigation of the molecular mechanisms of different types of variations, lists of genes (n_1_ = 3,177, n_2_ = 2,542, and n_3_ = 192) that overlapped with deletions, insertions and CNVs were submitted for IPA core analysis. The differentially expressed genes were categorized by related canonical pathways, disease and functional analyses and gene networks. For canonical pathways, the top enriched categories of canonical pathways for deletions with p values <0.05 as well as representative differentially expressed genes in each canonical pathway are listed in Supplementary Table S3. It was found that adenocarcinoma in the endometrium, breast or ovarian carcinoma, acute leukaemia, breast cancer and development of reproductive systems were significantly related. In addition to canonical pathways, differentially expressed genes were also categorized by related diseases and functions. Consistent with the results of canonical pathway analysis, the number of categories of diseases and functions increased at first and then progressively declined. Significantly activated functions were generally related to numerous diseases, especially reproductive system diseases and endocrine system disorders. In addition to the predominant pathways and cellular functions, gene networks have attracted much attention as they were built to connect key genes and enriched categories of diseases and functions based on the correlations between differentially expressed genes. For deletions, gene networks and their related top diseases and functions are presented in Table 5. The top networks of deletions have 24 pathways with scores >20. These top networks were mainly connected to the functions of lipid metabolism, reproductive system development and function, and embryonic development. Therefore, genes involved in these functional categories were further analysed. There were 35 genes in the 6th and 7th network, which were related to embryonic development and reproductive system development and function, respectively (Figure 3). It is interesting that some of the genes involved in different significant networks, such as core genes associated with embryonic development (e.g., CAMP responsive element binding protein 1 [*CREB1*] and retinoic acid receptor alpha, core genes related to reproductive system development and function (e.g., RAN binding protein 10 [*RANBP10*], required for meiotic nuclear division 5 homolog A [*RMND5A*], CREB binding protein), and eight core genes associated with lipid metabolism (e.g., HECT and RLD domain containing E3 ubiquitin protein ligase 2 [*HERC2*], nephrocystin 4, NIMA related kinase 4, *etc*.), play a key role in DNA and protein phosphorylation, acetylation, and ubiquitination, which are epigenetic processes (http://www.genecards.org). Thus, we can infer that these structural variations were mainly caused by environmental effects. For insertions, a total of 18 significant networks were associated with embryonic (e.g. SRSF protein kinase 2 [*SRPK2*], endothelial PAS domain protein 1 [*EPAS1*], etc.), immunological and inflammatory diseases (nuclear receptor subfamily 3 group C member 1, ligand dependent nuclear receptor interacting factor 1 [*LRIF1*], *etc*.) (Supplementary Table S4, Figure S3). Compared to deletions and insertions, the number of significant networks of CNVs was much smaller, but the types of significant diseases and functions were quite similar and related to inflammatory response (AKT serine/threonine kinase, inducible T cell costimulator, *etc*.) and lipid metabolism (amyloid beta precursor protein) (Supplementary Table S5, Figure S4). To sum up, the related networks of different types of variations are quite distinct in number and function. We combined all 102 genes associated with reproduction and embryonic development into a candidate gene set that is characterized by genes that cause reproduction problems in the Shanghai Holstein population, and all 48 genes associated with immunological and inflammatory diseases were combined into a candidate gene set that is characterized by genes that cause mastitis in Shanghai Holstein cattle.

## DISCUSSION

So far, structural variants in whole genomes have not been reported in the Shanghai Holstein population by using next-generation sequencing (NGS), and relatively few deletions, insertions and CNVs have been detected or confirmed. Shanghai Holstein cattle are the main economic animals providing milk, but due to the unique environment in Shanghai, which is characterize with hot weather condition and high humidity particularly in the summer with most days having temperature of above 35°C (95°F) and thereby subjecting them to many diseases as well as influencing the yield and quality of milk. Therefore, it is urgent to determine the mechanisms of structural variants related to these traits. In our study, we performed gene sequencing and structural variant calling in a large population, which was composed of 1,092 dairy cows from the Shanghai Bright Dairy and Food Co., Ltd. and the Dairy Cattle Breeding Centre born between 2001 and 2012. Furthermore the variants identified were annotated using bioinformatics tools.

A total of 1,092 samples were genotyped according to the GGRS protocol in our study. The average depth and coverage of the sequence data were 4.0% and 1.8%, respectively. A total of 16,831 deletions, 12,735 insertions and 490 CNVs from the sequence data of the Shanghai Holstein population were detected by using DELLY and cn.MOPS; among these CNVs, there were 102 CNVRs found in only one sample. Jiang et al [15] used the Illumina Bovine SNP50K Beadchip to screen 2,047 Chinese Holstein cattle, which were all collected from Beijing, and a total of 219, 169, and 140 CNVs were detected by PennCNV, GADA and cnvPartition, respectively [15]. In addition, the average size of these CNVs was 151.69 Kb. In our study, the distribution of deletions and insertions on each chromosome was quite uniform. However, distribution of CNVs was not uniform, which was consistent with the results obtained by using a SNP chip [33]. The average length of CNVs was 13,725 bp, which was much shorter than those based on the Bovine54K SNP chip. These results are consistent with the conclusion that CNVRs identified based on sequence data are the shortest [34]. The number of samples in our study was half that of the population used in the study done by Jiang [34], but the number of CNVs detected was much more.

SNP chip (Illumina Bovine SNP Beadchip) and high-throughput sequencing are both widely used for DNA sequencing of different animals. So far, the chips applied to bovines are the Illumina BovineSNP50 Beadchip with approximately 54K SNPs, BovineLD with approximately 7K SNPs and BovineHD with approximately 780,000 SNPs. In addition, SNP probes which cannot cover the whole genome on the chip are neither dense enough nor uniformly distributed to achieve an unbiased and high-resolution cattle CNV map. In addition, the price is relative expensive, and the reference population used to design the chips was composed of Europe and America cattle. Therefore, it is not completely appropriate for Asian populations. The other technology to study genetic variants is NGS, and since the advent of this technology, it has been rapidly evolving, with increasingly widespread adoption of several platforms and decreases in the cost of DNA sequencing, which allows for CNV reconstruction at a higher effective resolution and sensitivity and systematic identification CNVs at a genome-wide level [35]. Currently, these sequence-based approaches are becoming more popular due to ongoing developments. Accurate mapping and processing of NGS data are critical for analysis-ready reads and for downstream variant calling. This is the first time NGS methods have been used to detect CNVs in a large Shanghai Holstein population with low coverage, and it can be expected that a larger number of CNVs across genomes can be identified with this technique compared to the Bovine SNP50K Beadchip. Despite improvements to NGS technologies and CNV detecting tools, the identification of CNVs still remains a challenge. The numbers and size ranges of CNVs detected in different studies vary dramatically. In addition, the differences in sequence coverage, sample size, breed and CNV detection algorithms may be artefacts of these discrepancies [36].

Due to the importance of CNVs and other structural variations in the genome, advanced technologies have been created, including microarrays, that permit high-throughput methods that are now relatively common [37]. For identification of CNVs, which are an important source of genetic variation, there are several software tools available, such as cn.MOPS, CNVnator and Genome STRiP [29,35,38]. Genome STRiP is sufficient to detect deleted CNVs across the autosomes but does not have enough power to discover inserted events. Keel et al [36] gave us an appropriate strategy for detecting CNVs, which investigates the relationship between discovery power and coverage in CNV detection by comparing cn.MOPS, CNVnator and DELLY2 in sequences with varying levels of coverage using simulated CNV datasets. This study found that in all but the high coverage dataset, cn.MOPS and CNVnator had similar performance. In addition, because cn.MOPS models the depths of coverage across samples at each genomic position. Therefore, it does not suffer from read count biases along chromosomes. Thus, the stability of precision values for cn.MOPS is increased. The precision values of cn.MOPS were much better than that of CNVnator, whereas their recall values were comparable. In addition, CNVnator fails when the single copy length is lower than 2 kbp [39]. Therefore, in our study, we detected CNVs by using cn.MOPS, which is proposed to increase statistical power and decrease computational burden based on a multiple samples approach. cn.MOPS proposed a data processing pipeline using a mixture of Poisson models to reduce the FDR in CNV detection.

For deletions and insertions, at present, after quality control and recalibration, sequencing data are subsequently processed by different software such as DELLY, Breakdancer, SVseq2, CNNdel, MetaSV, and Pindel for calling SVs [27,40–44]. Generally, strategies for detecting SVs in NGS data have relied on four types of approaches, and read depth methods and assembly methods usually need data with greater coverage. Read pair methods and read depth methods are not able to find exact breakpoints of SVs. Split-read mapping methods may find exact breakpoints of some SVs with low-coverage data. The representative tools based on split reads are DELLY, SVseq2, and Pindel. Pindel is a very good choice for inversions at the size range where it operates (1 to 10 kbp) with sufficient coverage, and it is worth noting that it performs poorly under low coverage when calling SVs [41]. SVseq2 and DELLY are hybrid approaches to call SVs. DELLY realigns these split-reads to identify precise boundaries. The program predicts structural variants by taking into account the mapping of paired reads and local split read alignments. Although DELLY performs split-read analysis to refine its breakpoint predictions, it is not a required step of their algorithm for calling structural variants (i.e. DELLY can call SVs using only paired reads). And DELLY, used in our study, is also based on multiple samples. Moreover, compared with other tools, multiple sample-based tools often gain high sensitivity and a lower false positive rate [45]. There are many researches for comparing the performance of different tools with different depth, Nguyen et al [46] used 120 simulated paired-end samples with three types of depth of coverage (1–5×, 6–10×, 11–15×) found DELLY’s true positive rate was relatively high (>0.63) and had low FDRs with 1–5×. However, other packages had true positive rates less than 0.5 [46]. Kronenberg et al [47] compared the sensitivity and false discovery rates for simulated data of DELLY, LUMPY, and Wham, the results showed that all three tools exhibited a positive correlation between depth and FDR when comparing the 10× and 50× datasets. For example, DELLY’s FDR for deletions nearly doubles in the 50× relative to the 10× data [47].

By annotating the structural variants using GO terms and KEGG pathways, we found that many terms are significantly related to diseases and one of the reasons for this could be that the hot and high humid environment in Shanghai. For deletions, bta05200:Pathways in cancer, GO:0032845~negative regulation of homeostatic process, bta04142:Lysosome, GO: 0045321~leukocyte activation and bta05412:Arrhythmogenic right ventricular cardiomyopathy are all significant pathways, and among them, bta04142:Lysosome responds to foreign substances such as bacteria, viruses and other antigens. GO: 0045321~leukocyte activation is associated with mastitis [48]. For insertions, bta05200:Pathways in cancer, bta04062:Chemokine signalling pathway, bta04015:Rap1 signalling pathway, bta04670:Leukocyte transendothelial migration, bta05166: HTLV-I infection and bta04750:Inflammatory mediator regulation of TRP channels were reported in previous studies and result in different diseases, especially bta04750:Inflammatory mediator regulation of TRP channels, which exhibits a unique response to temperature. In addition, bta04670:Leukocyte transendothelial migration pathways are related to mastitis [49]. The bta04062:Chemokine signalling pathway is consistent with the phenomenon that more intense inflammation occurred in yellow cattle [50]. The pathway bta05166:HTLV-I infection is reported to be related to breast cancer; it is interesting that HTLV-1 is a delta retrovirus closely related to the bovine leukaemia virus, which is the most prevalent oncogenic virus of cattle, causing bovine leucosis and infecting mammary epithelial cells of cows; it may also be found in cow’s milk [51]. For CNVs, GO:0009628~response to abiotic stimulus is in any process that results in a change in state or activity of a cell or an organism (in terms of production, etc.) as a result of an abiotic (non-living) stimulus; thus, we can infer that the external environment, such as weather, management and artificial selection distinctively influenced the traits of the Shanghai Holstein cattle [52]. The most significant pathways are bta00240: Pyrimidine metabolism and bta00230:Purine metabolism, and both are essential pathways in animal and plant cells [53,54]. The IPA-derived gene network in deletions suggested that the categories of reproductive system development and functions, embryonic development and lipid metabolism were highly scored at 35, 35, and 30, respectively. In addition, many core genes were detected, such as *RANBP10*, which acts a novel coactivator for the androgen receptor [55]. Sallam et al [56] performed an across-breed (Holstein and Jersey) genome wide association analysis of susceptibility to paratuberculosis to identity the *CREB1* being associating with T-cell activation and interleukin-2 production [56]. *HERC2* was overlapped with the most significant core haplotypes in A. Gurgul’s identification of genome-wide selection signatures in the Limousin beef cattle breed [57]. Jaeger et al [58] provides first large-scale miRNA expression profiles and their predicted target genes in porcine mammary epithelial cells (PMECs), like *RMND5A* which identified a critical role of miRNAs in regulation of host immune responses and homeostasis of PMECs mediated by affecting pathways including cytokine binding [58]. In addition, for insertions in our study, the categories of endocrine system disorders, immunological and inflammatory disease and lipid metabolism were highly scored with value of 36, 34, and 29, respectively. An et al [52] reported that the endocrine plays crucial roles in a diverse set of developmental processes, as well as abiotic stresses. In addition, lipid metabolism influenced the milk fat production percentage [59]. Genes overlapped with insertions, involved in the gene-gene interaction network. It is noteworthy that *SRPK2* has been recognized as a differentially expressed gene between the endometrial tissue collected from day 7 of the estrous cycle of animals with high and low fertility by Aideen P Killeen’s microarray analysis [60]. Heaton et al [61] found that *EPAS1* associated with pulmonary hypertension in Angus cattle. Genetic variations of *NR3C2* could explain the alterations in animals to adapt to challenges, and therefore, their influence on production traits [62]. *LRIF1* is a nuclear protein that is known to be involved in the inactivation of the human X chromosome; however, its function in immunity is unknown [63]. Therefore, the results in our study indicate that the genetic mechanism of all the types of structural variations, deletions, insertions and CNVs are significantly associated with mastitis and reproductive obstacles.

Groenen et al [64] reported sequencing the whole genome of a Duroc pig by using next-generation analysis and building a map of gene sequences in 2012 [64]. In 2013, Li et al [65] sequenced a Tibetan pig and revealed the relationship between wild and domestic pigs and initially estimated that several genes were associated with disease [65]. However, there is no systematic analysis of the structural variants detection in Holstein cattle by NGS. Since the methods based on SNPs cannot detect complex regions effectively, many structural variants could not be detected. Therefore, the application of structural variants in genome-wide association study and functional annotations is heavily restricted.

So far, many studies have been conducted to discover CNVs; however, the association between CNVs and phenotype has not been analysed. Using genome-wide genotyping to discover the genetic variations is the only precondition for many studies, such as those investigating the molecular mechanisms of livestock breeding and conservation, especially for genome-wide association studies, genomic selection, and genomic conservation. In this study, we systematically identified structural variation, including deletions, insertions and CNVs and also determined the potential variations that may result in mastitis and affect reproduction. Therefore, for further studies, we will make full use of our findings in this study to estimate the genetic effect of structural variation for economic traits, gain a more particular knowledge of the population structure, and make decisions about the genomic selection programme. In addition, we aim to research on genomic predictions, thus laying a foundation for forming a reference population in Shanghai.

## Supplementary Data



## Figures and Tables

**Table 1:** Summary of average high-quality reads, depth and coverage of 12 libraries

Library ID	Individuals	Qualified reads	Depth	Coverage %
12	108	3,756,655	9.6	1.5
11	108	3,834,609	12.1	1.1
10	108	4,277,294	12.8	1.1
9	108	5,068,159	12.9	1.4
8	108	4,695,552	11.1	1.7
7	108	4,910,651	10.0	2.0
6	108	2,947,935	5.2	1.9
5	84	3,898,749	4.0	2.7
4	84	3,088,790	4.0	1.8
3	84	3,003,769	13.4	0.6
1–2	84	2,024,274	9.9	1.3

**Table 2:** The number (No.) and distribution of variants detected in each chromosome

Chr	Deletion			Insertion			Copy-number variants			Total No.3)
		
	No.1)	Genes2)	Ratio (%)	No. 1)	Genes2)	Ratio (%)	No. 1)	Genes2)	Ratio (%)	
1	245	147	14.92	184	123	12.49	6	6	0.61	985
2	226	145	14.20	171	114	11.17	9	12	1.18	1,021
3	278	172	12.54	207	135	9.84	8	8	0.58	1,372
4	262	145	16.96	200	117	13.68	9	10	1.17	855
5	239	147	11.11	197	126	9.52	7	7	0.53	1,323
6	192	110	15.90	148	89	12.86	6	7	1.01	692
7	205	147	10.53	146	102	7.31	14	15	1.07	1,396
8	180	113	13.63	133	92	11.10	5	5	0.60	829
9	160	92	15.28	120	78	12.96	6	6	1.00	602
10	257	166	15.46	193	133	12.38	6	7	0.65	1,074
11	231	144	13.75	170	121	11.56	9	9	0.86	1,047
12	95	61	14.73	76	51	12.32	4	4	0.97	414
13	203	144	16.94	149	115	13.53	7	7	0.82	850
14	134	77	13.49	102	63	11.03	1	1	0.18	571
15	159	101	9.62	108	75	7.14	4	4	0.38	1,050
16	169	107	15.07	127	82	11.55	8	8	1.13	710
17	136	84	12.63	89	59	8.87	11	12	1.80	665
18	160	110	8.90	124	90	7.28	12	16	1.29	1,236
19	233	144	10.69	161	112	8.31	12	13	0.97	1,347
20	108	64	16.67	90	57	14.84	0	0	0.00	384
21	140	85	11.63	103	67	9.17	9	9	1.23	731
22	204	105	17.27	158	86	14.14	7	7	1.15	608
23	124	75	9.55	94	63	8.03	3	4	0.51	785
24	122	63	18.16	95	56	16.14	7	7	2.02	347
25	144	87	11.36	80	55	7.18	23	35	4.57	766
26	136	78	17.85	103	66	15.10	5	5	1.14	437
27	67	43	15.69	55	39	14.23	1	1	0.36	274
28	128	69	19.44	103	56	15.77	4	4	1.13	355
29	108	69	9.79	74	51	7.23	2	3	0.43	705
X	119	66	5.85	91	59	5.23	6	7	0.62	1,128

1) The number of variations overlapping genes.

2) The total number of genes overlapping variations.

3) The number of genes on each chromosome.

**Table 3:** The description of variants in functional regions

Category	Deletion (%)	Insertion (%)	Copy-number variants (%)
Intergenic	11,662 (69.29)	8,882 (69.74)	279 (56.94)
Exonic	689 (4.09)	471 (3.70)	143 (29.18)
Intronic	4,354 (25.87)	3,304 (25.94)	8 (1.63)
Untranslated region	125 (0.74)	80 (0.63)	60 (12.24)
Total	16,830	12,735	490

**Table 4:** Distribution of variants among different traits

Trait	Deletion			Insertion			CNV		
		
	n1)	n2)	Genes3)	n1)	n2)	Genes3)	n1)	n2)	Genes3)
Exterior_QTL	826	254	164	628	192	128	18	6	10
Health_QTL	916	297	197	699	237	161	34	24	22
Meat_and_Carcass_QTL	5,361	1,826	1,120	4,061	1,362	898	126	75	72
Milk_QTL	5,724	2,004	1,233	4,299	1,457	973	160	85	82
Production_QTL	1,997	617	394	1,540	451	302	39	17	15
Reproduction_QTL	3,018	1,097	722	2,281	830	586	98	51	51
Exterior_Association	11	6	4	8	3	2	5	6	4
Health_Association	100	31	21	80	26	18	8	5	5
Meat_and_Carcass_Association	512	158	106	387	123	86	18	5	5
Milk_Association	75,356	20,877	12,159	60,490	15,764	10,274	807	207	207
Production_Association	44,649	11,895	7,228	35,784	9,074	6,067	446	178	176
Reproduction_Association	1,828	484	301	1,437	362	247	70	30	30

CNV, copy-number variants; QTL, quantitative trait locus.

1) The number of structure variants overlapping QTL regions.

2) The number of structure variants both overlapping QTL regions and genes.

3) The number of genes related to structure variations.

**Table 5:** The total ingenuity pathway analysis-derived gene networks for deletions

ID	Score	Focus molecules	Top diseases and functions
1	35	35	Post-Translational Modification, Connective Tissue Disorders, Developmental Disorder
2	35	35	Developmental Disorder, Hereditary Disorder, Ophthalmic Disease
3	35	35	Cellular Assembly and Organization, Cell Cycle, Haematological System Development and Function
4	35	35	Connective Tissue Development and Function, Connective Tissue Disorders, Organismal Injury and Abnormalities
5	35	35	Hereditary Disorder, Neurological Disease, Ophthalmic Disease
6	35	35	Embryonic Development, Endocrine System Development and Function, Organ Development
7	35	35	Reproductive System Development and Function, Cardiovascular System Development and Function, Embryonic Development
8	33	34	Cellular Assembly and Organization, Cellular Function and Maintenance, Cellular Movement
9	33	34	Cellular Function and Maintenance, Molecular Transport, Cellular Assembly and Organization
10	33	34	Cellular Compromise, Cellular Assembly and Organization, Cell Morphology
11	33	34	Amino Acid Metabolism, Molecular Transport, Small Molecule Biochemistry
12	33	34	Cancer, Cellular Development, Organismal Injury and Abnormalities
13	33	34	Cell-To-Cell Signalling and Interaction, Haematological System Development and Function, Cell Cycle
14	30	33	Cancer, Cellular Movement, Neurological Disease
15	30	33	Cell Cycle, DNA Replication, Recombination, and Repair, Cellular Development
16	30	33	Connective Tissue Disorders, Dermatological Diseases and Conditions, Developmental Disorder
17	30	33	Connective Tissue Disorders, Developmental Disorder, Hereditary Disorder
18	30	33	Post-Translational Modification, Cell Morphology, Infectious Diseases
19	30	33	Drug Metabolism, Glutathione Depletion in Liver, Developmental Disorder
20	30	33	Skeletal and Muscular System Development and Function, Digestive System Development and Function, Cell Morphology
21	30	33	Cell-To-Cell Signalling and Interaction, Haematological System Development and Function, Cellular Movement
22	30	33	Lipid Metabolism, Molecular Transport, Small Molecule Biochemistry
23	30	33	Lipid Metabolism, Small Molecule Biochemistry, Cellular Function and Maintenance
