# Supplementary material for: Detection of genome-wide structural variations in the Shanghai Holstein cattle population using next-generation sequencing
Source: Asian-Australas J Anim Sci. 2018 Jul 26;32(3):320–33. doi: 10.5713/ajas.18.0204 (PMC6409473; doi:10.5713/ajas.18.0204)
Supplement: Supplementary file 1 [file ajas-18-0204-supplementary.pdf]

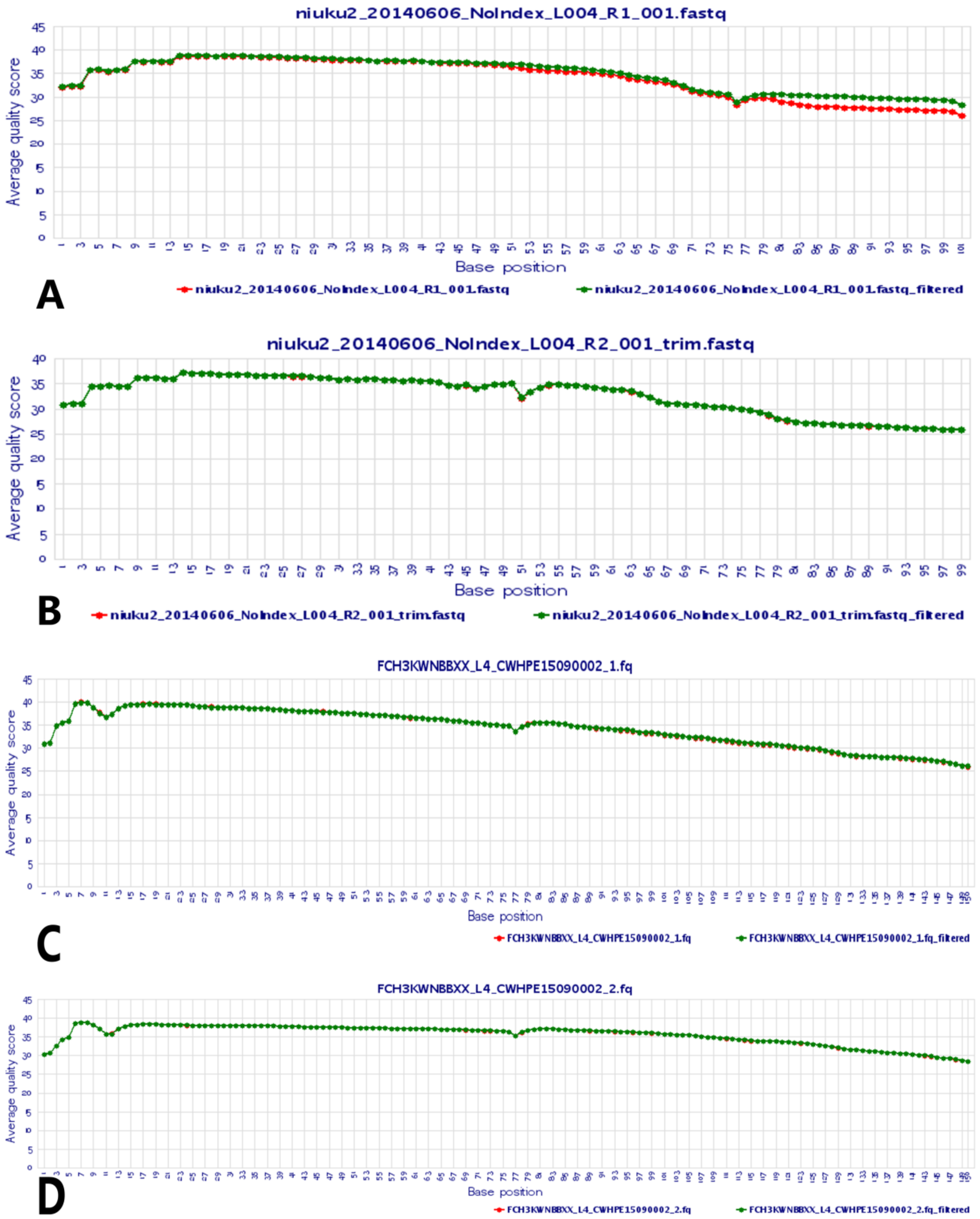

Supplementary Figure S1. The quality distribution of each base of the reads.

Note.The x-axis represents the base position, and the y-axis represents the score of the quality. The red line was the primary reads and the green line was the filter reads. A and B are the quality distribution for each base of the raw and filtered reads from R1 and R2 of library of 84. C and D are the quality distribution for each base of the raw and filtered reads from R1 and R2 of library of 108.

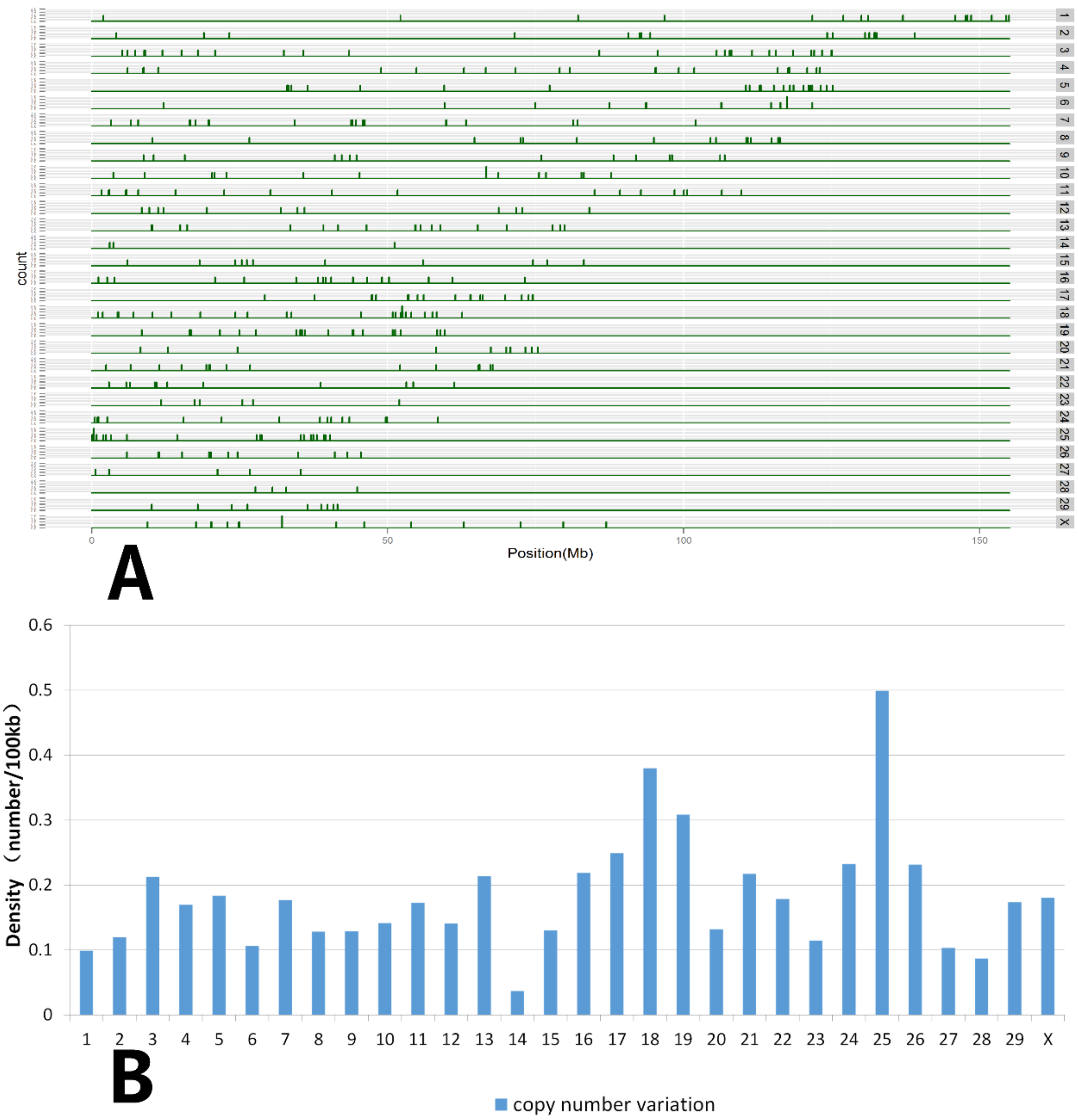

Supplementary Figure S2. Distribution of the density of CNVs across chromosomes.

Note. A.shows the location distribution of CNVs across the genome; B.demonstrates the distribution of the density of CNVs across chromosomes calculated as the number of structure variations per 100 kb.

Supplementary Table S1. The number and density distribution of insertion and deletion detected in each chromosome

CHR<br>**chromosome**	length<br>(bp)	insertion<br>Num[1]	 Density[1]	deletion<br>Num[2]	 Density[2]
1	158337067	7601	4.80052E-05	878	5.54513E-06
2	137060424	7381	5.38522E-05	836	4.74243E-06
3	121430405	7842	6.45802E-05	890	6.32461E-06
4	120829699	6296	5.21064E-05	748	6.70365E-06
5	121191424	6718	5.5433E-05	721	3.44084E-06
6	119458736	5127	4.29186E-05	628	4.08509E-06
7	112638659	6426	5.70497E-05	693	6.15242E-06
8	113384836	6336	5.58805E-05	730	6.43825E-06
9	105708250	5014	4.74324E-05	579	5.47734E-06
10	104305016	6014	5.76578E-05	650	6.23172E-06
11	107310763	6994	6.51752E-05	768	7.15678E-06
12	91163125	4046	4.4382E-05	810	8.88517E-06
13	84240350	6250	7.41925E-05	417	4.95012E-06
14	84648390	4398	5.19561E-05	488	5.76502E-06
15	85296676	4643	5.44335E-05	494	5.79155E-06
16	81724687	4793	5.86481E-05	528	6.46072E-06
17	75158596	4592	6.10975E-05	485	6.45302E-06
18	66004023	4924	7.46015E-05	493	7.46924E-06
19	64057457	5513	8.60634E-05	541	8.44554E-06
20	72042655	3947	5.4787E-05	432	5.99645E-06
21	71599096	4528	6.3241E-05	481	6.71796E-06
22	61435874	4090	6.65735E-05	447	7.27588E-06
23	52530062	3538	6.73519E-05	337	6.41537E-06
24	62714930	3573	5.69721E-05	397	6.33023E-06
25	42904170	3703	8.63086E-05	326	7.59833E-06
26	51681464	3089	5.977E-05	350	6.77225E-06
27	45407902	2771	6.10246E-05	284	6.25442E-06
28	46312546	3002	6.48204E-05	343	7.4062E-06
29	51505224	3426	6.65175E-05	381	7.39731E-06
**TOTAL**	2512082506	146575	5.8348E-05	17390	6.92254E-06

Supplementary Table S2. Significant GO terms and KEGG Pathways of deletion, insertion and CNVs.

Types	GO terms	annotation	Num	PValue
Deletion	GOTERM_MF_2	GO:0036094~small molecule binding	96	1.74E-05
	GOTERM_MF_2	GO:0097159~organic cyclic compound binding	176	2.91E-04
	GOTERM_MF_2	GO:1901363~heterocyclic compound binding	174	3.20E-04
	GOTERM_MF_2	GO:0097367~carbohydrate derivative binding	79	3.23E-04
	GOTERM_MF_2	GO:0005085~guanyl-nucleotide exchange factor activity	15	6.70E-04
	GOTERM_CC_2	GO:0005622~intracellular	353	7.24E-04
	GOTERM_CC_2	GO:0044446~intracellular organelle part	203	7.74E-04
	GOTERM_CC_2	GO:0044422~organelle part	207	7.77E-04
	KEGG_PATHWAY	bta05200:Pathways in cancer	23	1.21E-03
	GOTERM_BP_2	GO:0022402~cell cycle process	41	1.56E-03
	KEGG_PATHWAY	bta03410:Base excision repair	6	1.80E-03
	GOTERM_BP_2	GO:0016043~cellular component organization	163	1.81E-03
	GOTERM_BP_2	GO:0051641~cellular localization	74	1.84E-03
	GOTERM_MF_2	GO:0016787~hydrolase activity	83	3.92E-03
	GOTERM_CC_2	GO:0044424~intracellular part	335	4.69E-03
	KEGG_PATHWAY	bta02010:ABC transporters	6	5.29E-03
	GOTERM_CC_2	GO:0043228~non-membrane-bounded organelle	114	5.78E-03
	KEGG_PATHWAY	bta04110:Cell cycle	10	6.82E-03
	GOTERM_BP_2	GO:0032845~negative regulation of homeostatic process	10	8.61E-03
	GOTERM_MF_2	GO:0016740~transferase activity	76	8.90E-03
	KEGG_PATHWAY	bta04976:Bile secretion	7	1.01E-02
	KEGG_PATHWAY	bta04510:Focal adhesion	13	1.10E-02
	GOTERM_CC_2	GO:0043229~intracellular organelle	290	1.62E-02
	GOTERM_CC_2	GO:0044449~contractile fiber part	10	1.80E-02
	GOTERM_BP_2	GO:0032879~regulation of localization	67	1.85E-02
	GOTERM_CC_2	GO:0043233~organelle lumen	102	2.06E-02
	GOTERM_CC_2	GO:0043234~protein complex	119	2.10E-02
	KEGG_PATHWAY	bta04142:Lysosome	9	2.11E-02
	KEGG_PATHWAY	bta04611:Platelet activation	9	2.21E-02
	GOTERM_CC_2	GO:0044463~cell projection part	25	2.39E-02
	GOTERM_CC_2	GO:0098590~plasma membrane region	25	2.66E-02
	GOTERM_BP_2	GO:0048646~anatomical structure formation involved in morphogenesis	38	2.79E-02
	KEGG_PATHWAY	bta03013:RNA transport	10	2.90E-02
	KEGG_PATHWAY	bta04810:Regulation of actin cytoskeleton	12	3.03E-02
	GOTERM_BP_2	GO:0045321~leukocyte activation	25	3.16E-02
	GOTERM_CC_2	GO:0070161~anchoring junction	21	3.65E-02
	KEGG_PATHWAY	bta04520:Adherens junction	6	3.69E-02

	GOTERM_BP_2	GO:0033036~macromolecule localization	71	3.77E-02
	GOTERM_CC_2	GO:0044441~ciliary part	13	3.82E-02
	KEGG_PATHWAY	bta05412:Arrhythmogenic right ventricular cardiomyopathy (ARVC)	6	3.90E-02
	GOTERM_CC_2	GO:0005929~cilium	18	3.99E-02
	GOTERM_BP_2	GO:0009653~anatomical structure morphogenesis	73	4.22E-02
	GOTERM_BP_2	GO:0044085~cellular component biogenesis	74	4.46E-02
insertion	KEGG_PATHWAY	bta05200:Pathways in cancer	20	2.05E-04
	GOTERM_BP_2	GO:0022402~cell cycle process	32	1.15E-03
	GOTERM_CC_2	GO:0044422~organelle part	149	1.15E-03
	GOTERM_CC_2	GO:0044446~intracellular organelle part	145	1.80E-03
	GOTERM_CC_2	GO:0043228~non-membrane-bounded organelle	86	2.05E-03
	GOTERM_MF_2	GO:0036094~small molecule binding	63	2.29E-03
	GOTERM_MF_2	GO:0005085~guanyl-nucleotide exchange factor activity	11	3.14E-03
	GOTERM_MF_2	GO:1901363~heterocyclic compound binding	119	4.86E-03
	KEGG_PATHWAY	bta04510:Focal adhesion	11	6.41E-03
	GOTERM_MF_2	GO:0097367~carbohydrate derivative binding	53	6.54E-03
	GOTERM_MF_2	GO:0097159~organic cyclic compound binding	119	7.51E-03
	GOTERM_CC_2	GO:0005622~intracellular	246	7.78E-03
	KEGG_PATHWAY	bta04062:Chemokine signaling pathway	10	8.87E-03
	KEGG_PATHWAY	bta04611:Platelet activation	8	1.07E-02
	GOTERM_MF_2	GO:0016740~transferase activity	55	1.14E-02
	GOTERM_BP_2	GO:0048646~anatomical structure formation involved in morphogenesis	30	1.39E-02
	KEGG_PATHWAY	bta04810:Regulation of actin cytoskeleton	10	2.07E-02
	KEGG_PATHWAY	bta04015:Rap1 signaling pathway	10	2.13E-02
	GOTERM_CC_2	GO:0044449~contractile fiber part	8	2.15E-02
	GOTERM_CC_2	GO:0044424~intracellular part	234	2.17E-02
	KEGG_PATHWAY	bta03410:Base excision repair	4	2.46E-02
	KEGG_PATHWAY	bta04670:Leukocyte transendothelial migration	7	2.62E-02
	GOTERM_MF_2	GO:0043167~ion binding	78	2.71E-02
	GOTERM_BP_2	GO:0016043~cellular component organization	111	2.84E-02
	KEGG_PATHWAY	bta04110:Cell cycle	7	3.23E-02
	KEGG_PATHWAY	bta05166:HTLV-I infection	11	3.31E-02
	GOTERM_CC_2	GO:0043229~intracellular organelle	204	3.35E-02
	GOTERM_BP_2	GO:0032845~negative regulation of homeostatic process	7	3.77E-02
	GOTERM_CC_2	GO:0070161~anchoring junction	16	3.94E-02
	KEGG_PATHWAY	bta05205:Proteoglycans in cancer	9	4.05E-02
	GOTERM_MF_2	GO:0016787~hydrolase activity	55	4.06E-02
	KEGG_PATHWAY	bta04976:Bile secretion	5	4.07E-02
	KEGG_PATHWAY	bta04022:cGMP-PKG signaling pathway	8	4.07E-02
	GOTERM_MF_2	GO:0022857~transmembrane transporter activity	27	4.16E-02

	KEGG_PATHWAY	bta02010:ABC transporters	4	4.57E-02
	GOTERM_BP_2	GO:0051641~cellular localization	48	4.67E-02
	KEGG_PATHWAY	bta04530:Tight junction	7	4.72E-02
	GOTERM_BP_2	GO:0040012~regulation of locomotion	19	4.75E-02
	GOTERM_CC_2	GO:0044441~ciliary part	10	4.76E-02
	GOTERM_BP_2	GO:0044085~cellular component biogenesis	54	4.80E-02
	KEGG_PATHWAY	bta04750:Inflammatory mediator regulation of TRP channels	6	4.87E-02
	GOTERM_BP_2	GO:0032259~methylation	10	4.98E-02
CNVs	KEGG_PATHWAY	bta00240:Pyrimidine metabolism	5	0.004919
	GOTERM_MF_2	GO:0036094~small molecule binding	24	0.013249
	GOTERM_CC_2	GO:0097458~neuron part	12	0.021552
	GOTERM_CC_2	GO:0043234~protein complex	34	0.027186
	KEGG_PATHWAY	bta00230:Purine metabolism	5	0.032003
	GOTERM_CC_2	GO:0043228~non-membrane-bounded organelle	31	0.037423
	GOTERM_CC_2	GO:0044422~organelle part	52	0.041734
	GOTERM_BP_2	GO:0051234~establishment of localization	31	0.053809
	GOTERM_CC_2	GO:0042995~cell projection	14	0.058477
	GOTERM_BP_2	GO:0051051~negative regulation of transport	6	0.060022
	GOTERM_BP_2	GO:0022402~cell cycle process	11	0.067766
	GOTERM_BP_2	GO:0009628~response to abiotic stimulus	9	0.068197
	GOTERM_CC_2	GO:0099572~postsynaptic specialization	3	0.075246
	GOTERM_CC_2	GO:0044463~cell projection part	8	0.081632
	GOTERM_CC_2	GO:0060076~excitatory synapse	3	0.094223
	GOTERM_MF_2	GO:0097367~carbohydrate derivative binding	18	0.096216
	GOTERM_BP_2	GO:1904950~negative regulation of establishment of protein localization	4	0.097693

Supplementary Table S3. Pathways from the Ingenuity Pathways Analysis library of canonical pathways that were most significant to the dataset for deletions.

**Categories**	**Diseases or Functions Annotation**	**p-Value**	**Molecules**
Cancer, Organismal Injury and Abnormalities, Reproductive System Disease	female genital tract adenocarcinoma	3.57E-72	1106
Cancer, Organismal Injury and Abnormalities, Reproductive System Disease	female genital neoplasm	1.41E-68	1241
Cancer, Organismal Injury and Abnormalities, Reproductive System Disease	female genital tract cancer	1.67E-65	1194
Cancer, Organismal Injury and Abnormalities, Reproductive System Disease	adenocarcinoma in endometrium	2.80E-64	853
Organismal Injury and Abnormalities, Reproductive System Disease	abnormality of endometrium	6.21E-64	912
Cancer, Organismal Injury and Abnormalities	breast or colorectal cancer	2.37E-51	1571
Cancer, Organismal Injury and Abnormalities	breast or ovarian carcinoma	5.88E-36	645
Cancer, Organismal Injury and Abnormalities, Reproductive System Disease	breast or ovarian cancer	7.06E-31	794
Cancer, Endocrine System Disorders, Organismal Injury and Abnormalities, Reproductive System Disease	epithelial ovarian cancer	3.15E-29	446
Cancer, Endocrine System Disorders, Organismal Injury and Abnormalities, Reproductive System Disease	adenosquamous ovarian cancer	5.14E-20	296
Cancer, Hematological Disease, Immunological Disease, Organismal Injury and Abnormalities	acute leukemia	1.19E-14	354
Cancer, Organismal Injury and Abnormalities, Reproductive System Disease	carcinoma in breast	2.19E-13	284
Cancer, Hematological Disease, Immunological Disease, Organismal Injury and Abnormalities	acute myeloid leukemia	3.90E-13	315
Cancer, Organismal Injury and Abnormalities, Reproductive System Disease	breast cancer	2.14E-11	441
Cancer, Hematological Disease, Immunological Disease, Organismal Injury and Abnormalities	chronic leukemia	4.87E-10	156
Cancer, Organismal Injury and Abnormalities, Reproductive System Disease	benign neoplasm of female genital organ	1.82E-09	139
Organismal Development, Reproductive System Development and Function	development of reproductive system	3.12E-07	204
Cancer, Organismal Injury and Abnormalities, Reproductive System Disease	female genital tract serous carcinoma	1.01E-06	116
Cancer, Organismal Injury and Abnormalities	embryonal tumor	1.24E-06	103
Cancer, Organismal Injury and Abnormalities	adenoma	1.57E-06	159
Cancer, Hematological Disease, Immunological Disease, Organismal Injury and Abnormalities	acute lymphocytic leukemia	1.98E-06	83

Cellular Development, Cellular Growth and Proliferation, Embryonic Development	proliferation of embryonic cell lines	9.52E-06	49

Supplementary Table S4. The summary of significant networks for insertions were generated through the use of Ingenuity Pathways Analysis. The network contained 18 of 25 focus molecules with a score of 30. A score of 30 means there is a 1 in 10^{30} chance of this network occurring by random chance.

ID	Score	Focus Molecules	Top Diseases and Functions
1	38	35	Amino Acid Metabolism, Post-Translational Modification, Small Molecule Biochemistry
2	38	35	Hereditary Disorder, Neurological Disease, Organismal Injury and Abnormalities
3	38	35	Gastrointestinal Disease, Hepatic System Disease, Organismal Injury and Abnormalities
4	36	34	Organismal Injury and Abnormalities, Cellular Development, Connective Tissue Development and Function
5	36	34	Cellular Assembly and Organization, Cell Cycle, Cellular Development
6	36	34	Organ Morphology, Organismal Development, Organismal Injury and Abnormalities
7	36	34	Cell Morphology, Organ Morphology, Organismal Development
8	36	34	Cardiac Arrythmia, Cardiovascular Disease, Organismal Injury and Abnormalities
9	36	34	Endocrine System Disorders, Hematological Disease, Metabolic Disease
10	34	33	Hereditary Disorder, Neurological Disease, Organismal Injury and Abnormalities
11	34	33	Connective Tissue Disorders, Immunological Disease, Inflammatory Disease
12	34	33	Connective Tissue Development and Function, Skeletal and Muscular System Development and Function, Tissue Development
13	34	33	Cellular Assembly and Organization, Auditory Disease, Cancer
14	34	33	Cellular Assembly and Organization, Cellular Development, Carbohydrate Metabolism
15	31	32	Cellular Development, Cellular Growth and Proliferation, Cancer
16	31	32	Cardiovascular System Development and Function, Embryonic Development, Organismal Development
17	31	32	Cardiovascular Disease, Cell Death and Survival, Connective Tissue Disorders
18	31	32	Amino Acid Metabolism, Small Molecule Biochemistry, Gastrointestinal Disease
19	29	31	Cell Cycle, Cell Morphology, Cellular Assembly and Organization
20	29	31	Cell-To-Cell Signaling and Interaction, Hematological System Development and Function, Cell Morphology
21	29	31	Cardiovascular Disease, Hereditary Disorder, Organismal Injury and Abnormalities
22	29	31	Lipid Metabolism, Molecular Transport, Small Molecule Biochemistry
23	29	31	Post-Translational Modification, Cellular Development, Cellular Growth and Proliferation
24	29	31	Cardiovascular Disease, Cardiovascular System Development and Function, Organ Morphology
25	29	31	Hereditary Disorder, Organismal Injury and Abnormalities, Skeletal and Muscular Disorders

Supplementary Table S5. The summary of significant networks for CNVs were generated through the use of Ingenuity Pathways Analysis.

ID	Score	Focus Molecules	Top Diseases and Functions
1	33	19	Cell Signaling, Molecular Transport, Nucleic Acid Metabolism
2	31	18	Cancer, Gastrointestinal Disease, Organismal Injury and Abnormalities
3	29	17	Nucleic Acid Metabolism, Small Molecule Biochemistry, Developmental Disorder
4	26	16	Cancer, Neurological Disease, Organismal Injury and Abnormalities
5	24	15	Inflammatory Response, Cellular Development, Cellular Growth and Proliferation
6	24	15	Cellular Assembly and Organization, Nervous System Development and Function, Tissue Morphology
7	22	14	Lipid Metabolism, Nucleic Acid Metabolism, Small Molecule Biochemistry

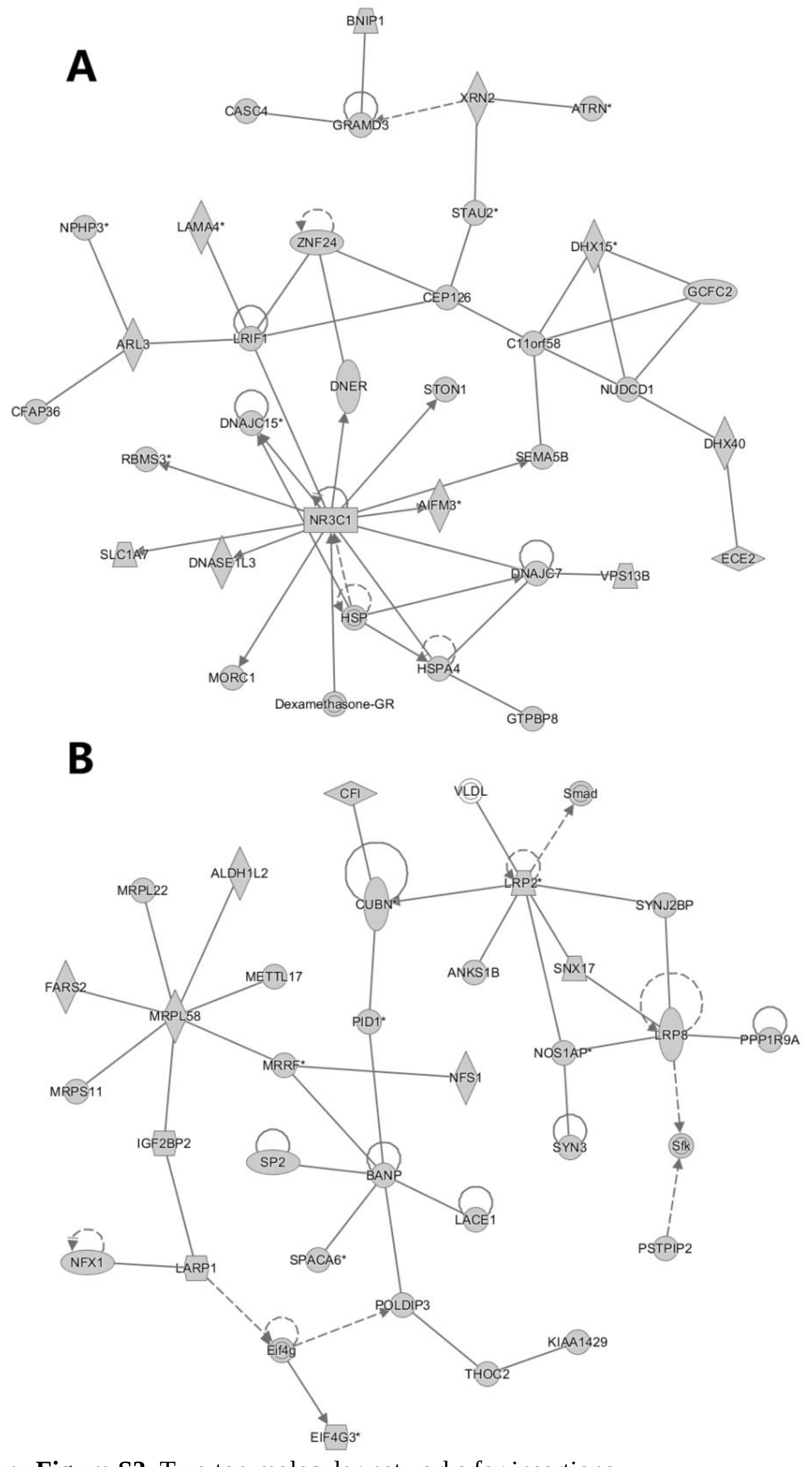

Supplementary Figure S3. Two top molecular networks for insertions.

Note. Gene network analysis by insertions calling by DELLY. A. shows the network significant related to Immunological Disease and Inflammatory Disease. B. shows the network significant associated with Lipid Metabolism. Solid graphic represents the genes in original module, hollow graphic represents the genes or biomolecules which add to the network from IPA database to complete the whole network, and the full line shows the direct interaction between genes, and the imaginary line shows the indirect interaction between genes.

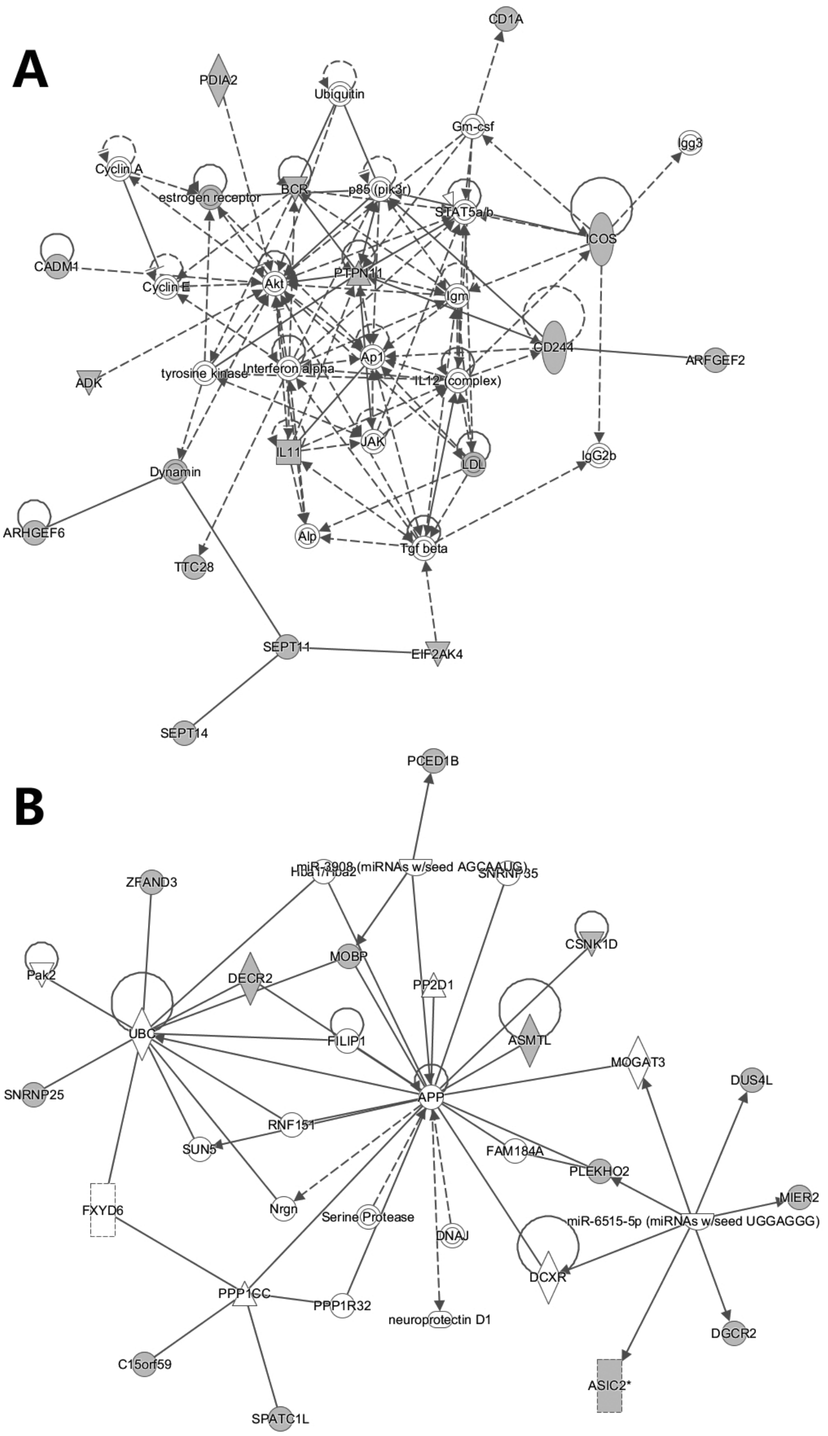

Supplementary Figure S4. Two top molecular networks for CNVs.

Note. Gene network analysis by CNVs calling by cn.MOPS. A. shows the network significant related to Inflammatory Response. B. shows the network significant associated with Lipid Metabolism. Solid graphic represents the genes in original module, hollow graphic represents the genes or biomolecules which add to the network from IPA database to complete the whole network, and the full line shows the direct interaction between genes, and the imaginary line shows the indirect interaction between genes.
